# 2,5-Dimethyl-*N*-[1-(1*H*-pyrrol-2-yl)ethyl­idene]aniline

**DOI:** 10.1107/S1600536812040858

**Published:** 2012-10-06

**Authors:** Bi-Yun Su, Wen-Long Qin, Jia-Xiang Wang

**Affiliations:** a College of Chemistry and Chemical Engineering, Xi’an ShiYou University, Xi’an, Shaanxi 710065, People’s Republic of China; b College of Petroleum Engineering, Xi’an ShiYou University, Xi’an, Shaanxi 710065, People’s Republic of China

## Abstract

In the title compound, C_14_H_16_N_2_, the pyrrole and benzene rings form a dihedral angle of 72.37 (8)°. In the crystal, centrosymmetrically related mol­ecules are assembled into dimers by by pairs of N—H⋯N hydrogen bonds, generating rings of *R*_2_^2^(10) graph-set motif. C—H⋯π inter­actions also occur.

## Related literature

For general background to the imino­pyrrole unit, see: Britovsek *et al.* (2003 ▶); Dawson *et al.* (2000 ▶); Wu *et al.* (2003 ▶). For the pyrrole diimine unit, see: Matsuo *et al.* (2001 ▶) and for the pyrrole monoimine unit, see: He *et al.* (2009 ▶); Su *et al.* (2009*a* ▶,*b* ▶). 
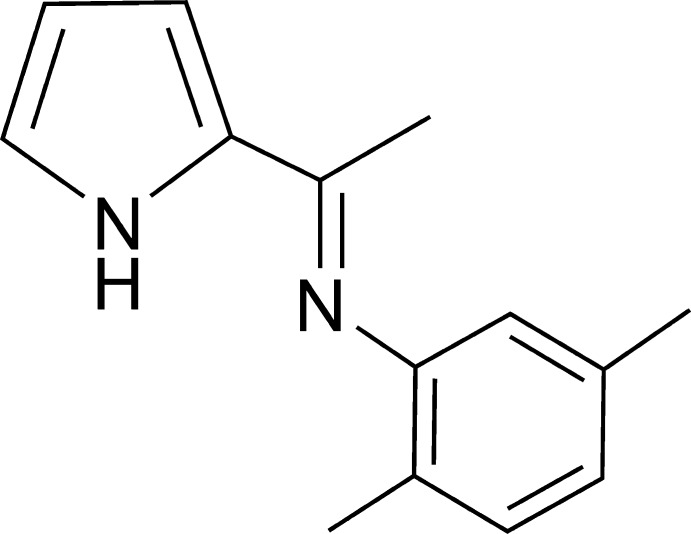


## Experimental

### 

#### Crystal data

C_14_H_16_N_2_
*M* *_r_* = 212.29
Monoclinic, 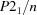

*a* = 12.5894 (17) Å
*b* = 7.3109 (10) Å
*c* = 14.8425 (19) Å
β = 113.118 (2)°
*V* = 1256.4 (3) Å^3^
*Z* = 4
Mo *K*α radiation
μ = 0.07 mm^−1^
*T* = 296 K
0.37 × 0.28 × 0.15 mm

#### Data collection

Bruker APEXII CCD diffractometer
Absorption correction: multi-scan (*SADABS*; Bruker, 2008 ▶) *T*_min_ = 0.976, *T*_max_ = 0.990
6463 measured reflections
2441 independent reflections
1885 reflections with *I* > 2σ(*I*)
*R*_int_ = 0.021

#### Refinement

*R*[*F* ^2^ > 2σ(*F* ^2^)] = 0.045
*wR*(*F* ^2^) = 0.156
*S* = 0.96
2441 reflections
149 parameters
H-atom parameters constrained
Δρ_max_ = 0.22 e Å^−3^
Δρ_min_ = −0.16 e Å^−3^

### 

Data collection: *APEX2* (Bruker,2008 ▶); cell refinement: *SAINT* (Bruker,2008 ▶); data reduction: *SAINT*; program(s) used to solve structure: *SHELXS97* (Sheldrick, 2008 ▶); program(s) used to refine structure: *SHELXL97* (Sheldrick, 2008 ▶); molecular graphics: *SHELXTL* (Sheldrick, 2008 ▶); software used to prepare material for publication: *publCIF* (Westrip, 2010 ▶).

## Supplementary Material

Crystal structure: contains datablock(s) I, global. DOI: 10.1107/S1600536812040858/rz5010sup1.cif

Structure factors: contains datablock(s) I. DOI: 10.1107/S1600536812040858/rz5010Isup2.hkl

Supplementary material file. DOI: 10.1107/S1600536812040858/rz5010Isup3.cml

Additional supplementary materials:  crystallographic information; 3D view; checkCIF report

## Figures and Tables

**Table 1:** Hydrogen-bond geometry (Å, °) *Cg1* is the centroid of the C7–C12 ring.

*D*—H⋯*A*	*D*—H	H⋯*A*	*D*⋯*A*	*D*—H⋯*A*
N1—H1⋯N2^i^	0.86	2.34	3.1354 (18)	155
C1—H1*A*⋯*Cg*1^i^	0.93	2.65	3.4298 (16)	142

Symmetry code: (i) 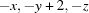.

**Fig. 1.**
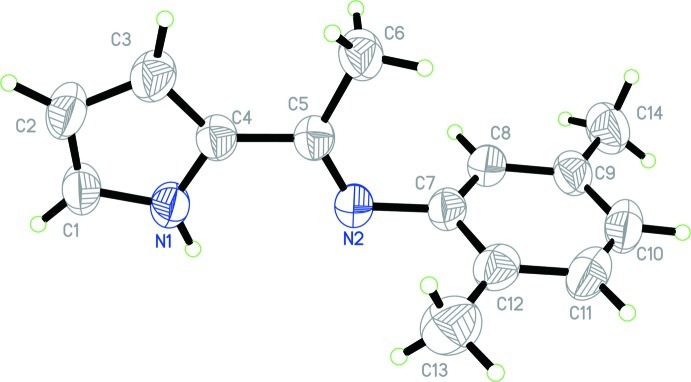
The molecular structure of the title compound. Displacement ellipsoids are drawn at the 50% probability level. H atoms are presented as small spheres of arbitrary radius.

**Fig. 2.**
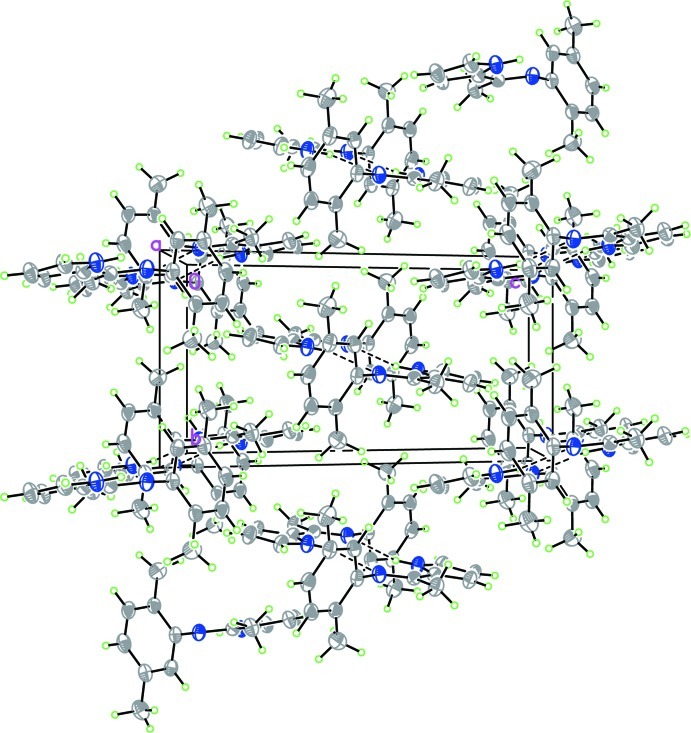
The crystal packing of the title compound viewed down the a axis, with hydrogen bond is shown as dashed lines.

## supplementary crystallographic information

### Comment

Bis(imino)pyrrole is usually prepared from Schiff bases condensation of 2,5-diacetylpyrrole and the aromatic amine (Matsuo *et al.*, 2001). Schiff bases containing pyrrole units have been extensively investigated because of their excellent and flexible coordination abilities (Wu *et al.*, 2003). As the five-memberd ring substitute of pyridine six-memberd ring (Matsuo *et al.*, 2001; He *et al.*, 2009), pyrrole has been frequently introduced into the skeleton of the bis(imino)pyridine ligand to design new ligands and corresponding metal complexes as catalysts of olefin polymerizations (Britovsek *et al.*, 2003; Dawson *et al.*, 2000). As a part of our studies on mono(imino)pyrrole ligands (Su *et al.*, 2009*a*,*b*), the crystal structure of the title compound is reported here.

The X-ray analysis of the title compound (Fig. 1) shows that the molecule is non-planar, with a dihedral angle of 72.37 (8)° formed by the pyrrole and benzene rings. The imino N—C bond length (1.288 (2) Å) indicates a C═N double bond character. In the crystal (Fig. 2), a pair of classical N–H···N hydrogen bonds (Table 1) link centrosymmetrically related molecules into a dimer, generating a ring of *R*_2_^2^(10) graph-set motif. The dimer is further enforced by C—H···π hydrogen interactions.

### Experimental

The reagents 2-acetyl pyrrole (0.1528 g, 1.40 mmol) and 2,5-dimethylaniline (0.3393 g, 2.80 mmol) were placed in a 50-ml flask. A few drops of acetic acid was then added in, and the mixture was subjected to radiation in a 800 W microwave oven for 3 min and 2 min on a medium–heat setting. The reaction was monitored by TLC, and the crude product was purified by silica gel column chromatography (eluant: petroleum ether/ethyl acetate, 5:1 *v*/*v*). Plate-like colourless single crystals used in X-ray diffraction studies were grown from an ethanolic solution by slow evaporation of the solvent at room temperature; yield 72.79%, 0.2982 g. *M*.p. 396.8–398.4 K. The purity and the composition of the compound were checked and characterized by IR, ^1^H NMR, mass spectrum, as well as elemental analysis. IR (KBr): ν_C=N_ 1659 cm^-1^. ^1^H NMR (400 MHz, CDCl_3_): δ 7.12 (d, 1H, benzene ring aromatic H), δ 7.09 (d, 1H, benzene ring aromatic H), 6.88 (m, 1H, benzene ring aromatic H), 6.56 (d, 1H, pyrrole ring aromatic H), 6.37 (s, 1H, pyrrole ring aromatic H), 6.18 (d, 1H, pyrrole ring aromatic H), 2.18 (s, 6H, phenyl-CH_3_), 2.05 (s, 3H, –N=C(CH_3_)-). MS (EI): m/*z* 212 (*M*). Anal. Calcd. for C_14_H_16_N_2_: C, 79.21; H, 7.60; N, 13.20. Found: C, 79.72; H, 7.13; N, 12.84.

### Refinement

All H atoms were placed at calculated positions and refined as riding, with C—H = 0.93–0.96 Å, N—H = 0.86 Å, and with *U*_iso_(H) = 1.2 *U*_eq_(C, N) or 1.5 *U*_eq_(C) for methyl H atoms.

### Crystal data

**Table:**

C_14_H_16_N_2_	*F*(000) = 456
*M**_r_* = 212.29	*D*_x_ = 1.122 Mg m^−^^3^
Monoclinic, *P*2_1_/*n*	Mo *K*α radiation, λ = 0.71073 Å
Hall symbol: -P 2yn	Cell parameters from 2093 reflections
*a* = 12.5894 (17) Å	θ = 2.7–26.0°
*b* = 7.3109 (10) Å	µ = 0.07 mm^−^^1^
*c* = 14.8425 (19) Å	*T* = 296 K
β = 113.118 (2)°	Block, colourless
*V* = 1256.4 (3) Å^3^	0.37 × 0.28 × 0.15 mm
*Z* = 4	

### Data collection

**Table:**

Bruker APEXII CCD diffractometer	2441 independent reflections
Radiation source: fine-focus sealed tube	1885 reflections with *I* > 2σ(*I*)
Graphite monochromator	*R*_int_ = 0.021
φ and ω scans	θ_max_ = 26.0°, θ_min_ = 2.7°
Absorption correction: multi-scan (*SADABS*; Bruker, 2008)	*h* = −15→13
*T*_min_ = 0.976, *T*_max_ = 0.990	*k* = −8→8
6463 measured reflections	*l* = −18→15

### Refinement

**Table:**

Refinement on *F*^2^	Secondary atom site location: difference Fourier map
Least-squares matrix: full	Hydrogen site location: inferred from neighbouring sites
*R*[*F*^2^ > 2σ(*F*^2^)] = 0.045	H-atom parameters constrained
*wR*(*F*^2^) = 0.156	*w* = 1/[σ^2^(*F*_o_^2^) + (0.1*P*)^2^ + 0.1684*P*] where *P* = (*F*_o_^2^ + 2*F*_c_^2^)/3
*S* = 0.96	(Δ/σ)_max_ = 0.001
2441 reflections	Δρ_max_ = 0.22 e Å^−^^3^
149 parameters	Δρ_min_ = −0.16 e Å^−^^3^
0 restraints	Extinction correction: *SHELXL*, Fc^*^=kFc[1+0.001xFc^2^λ^3^/sin(2θ)]^-1/4^
Primary atom site location: structure-invariant direct methods	Extinction coefficient: 0.030 (5)

### Special details

**Table:**

Geometry. All e.s.d.'s (except the e.s.d. in the dihedral angle between two l.s. planes) are estimated using the full covariance matrix. The cell e.s.d.'s are taken into account individually in the estimation of e.s.d.'s in distances, angles and torsion angles; correlations between e.s.d.'s in cell parameters are only used when they are defined by crystal symmetry. An approximate (isotropic) treatment of cell e.s.d.'s is used for estimating e.s.d.'s involving l.s. planes.
Refinement. Refinement of *F*^2^ against ALL reflections. The weighted *R*-factor *wR* and goodness of fit *S* are based on *F*^2^, conventional *R*-factors *R* are based on *F*, with *F* set to zero for negative *F*^2^. The threshold expression of *F*^2^ > σ(*F*^2^) is used only for calculating *R*-factors(gt) *etc*. and is not relevant to the choice of reflections for refinement. *R*-factors based on *F*^2^ are statistically about twice as large as those based on *F*, and *R*- factors based on ALL data will be even larger.

### Fractional atomic coordinates and isotropic or equivalent isotropic displacement parameters (Å^2^)

**Table:**

	*x*	*y*	*z*	*U*_iso_*/*U*_eq_	
N1	0.02765 (11)	0.93936 (18)	0.15136 (9)	0.0509 (4)	
H1	−0.0111	0.9621	0.0903	0.061*	
N2	0.16704 (11)	0.92217 (17)	0.04350 (9)	0.0493 (4)	
C1	−0.01726 (14)	0.9302 (2)	0.22049 (12)	0.0556 (5)	
H1A	−0.0944	0.9481	0.2098	0.067*	
C2	0.06832 (16)	0.8908 (2)	0.30727 (13)	0.0606 (5)	
H2	0.0608	0.8759	0.3667	0.073*	
C3	0.17118 (15)	0.8764 (2)	0.29152 (12)	0.0572 (5)	
H3	0.2441	0.8510	0.3388	0.069*	
C4	0.14446 (13)	0.90679 (19)	0.19355 (11)	0.0447 (4)	
C5	0.21617 (12)	0.90262 (18)	0.13711 (11)	0.0429 (4)	
C6	0.34366 (13)	0.8754 (3)	0.19343 (12)	0.0584 (5)	
H6A	0.3790	0.8420	0.1491	0.088*	
H6B	0.3559	0.7799	0.2409	0.088*	
H6C	0.3774	0.9870	0.2263	0.088*	
C7	0.23363 (12)	0.9170 (2)	−0.01501 (11)	0.0463 (4)	
C8	0.29383 (12)	1.0713 (2)	−0.02269 (11)	0.0495 (4)	
H8	0.2956	1.1732	0.0154	0.059*	
C9	0.35159 (13)	1.0782 (2)	−0.08547 (12)	0.0519 (4)	
C10	0.34733 (15)	0.9243 (2)	−0.14132 (12)	0.0593 (5)	
H10	0.3852	0.9247	−0.1840	0.071*	
C11	0.28748 (15)	0.7705 (2)	−0.13432 (12)	0.0596 (5)	
H11	0.2865	0.6689	−0.1723	0.072*	
C12	0.22837 (14)	0.7620 (2)	−0.07228 (11)	0.0519 (4)	
C13	0.41634 (15)	1.2478 (3)	−0.09201 (14)	0.0697 (6)	
H13A	0.4961	1.2359	−0.0489	0.105*	
H13B	0.3837	1.3525	−0.0733	0.105*	
H13C	0.4104	1.2630	−0.1581	0.105*	
C14	0.16000 (19)	0.5964 (3)	−0.06737 (16)	0.0763 (6)	
H14A	0.1561	0.5120	−0.1181	0.115*	
H14B	0.0833	0.6330	−0.0762	0.115*	
H14C	0.1969	0.5386	−0.0047	0.115*	

### Atomic displacement parameters (Å^2^)

**Table:**

	*U*^11^	*U*^22^	*U*^33^	*U*^12^	*U*^13^	*U*^23^
N1	0.0432 (7)	0.0719 (9)	0.0400 (7)	0.0059 (6)	0.0191 (6)	0.0070 (6)
N2	0.0423 (7)	0.0664 (9)	0.0424 (7)	0.0002 (6)	0.0202 (6)	−0.0022 (6)
C1	0.0489 (9)	0.0738 (11)	0.0523 (10)	0.0075 (8)	0.0288 (8)	0.0091 (8)
C2	0.0652 (10)	0.0790 (12)	0.0473 (9)	0.0149 (9)	0.0326 (8)	0.0163 (8)
C3	0.0541 (9)	0.0734 (11)	0.0453 (9)	0.0149 (8)	0.0209 (7)	0.0138 (8)
C4	0.0438 (8)	0.0480 (8)	0.0448 (8)	0.0050 (6)	0.0201 (7)	0.0048 (6)
C5	0.0433 (8)	0.0435 (8)	0.0443 (8)	0.0028 (6)	0.0199 (7)	0.0018 (6)
C6	0.0464 (9)	0.0780 (11)	0.0532 (10)	0.0155 (8)	0.0224 (8)	0.0126 (8)
C7	0.0386 (7)	0.0633 (10)	0.0379 (8)	0.0077 (6)	0.0160 (6)	0.0014 (6)
C8	0.0434 (8)	0.0631 (10)	0.0434 (9)	0.0018 (7)	0.0186 (7)	−0.0037 (7)
C9	0.0407 (8)	0.0717 (11)	0.0447 (9)	0.0111 (7)	0.0182 (7)	0.0096 (7)
C10	0.0574 (10)	0.0814 (13)	0.0470 (9)	0.0206 (9)	0.0290 (8)	0.0107 (8)
C11	0.0686 (11)	0.0677 (11)	0.0442 (9)	0.0206 (9)	0.0240 (8)	−0.0019 (7)
C12	0.0515 (9)	0.0592 (10)	0.0417 (8)	0.0093 (7)	0.0146 (7)	0.0018 (7)
C13	0.0578 (10)	0.0887 (14)	0.0699 (12)	−0.0006 (9)	0.0330 (9)	0.0152 (10)
C14	0.0877 (14)	0.0680 (13)	0.0735 (14)	−0.0043 (10)	0.0318 (12)	−0.0063 (9)

### Geometric parameters (Å, º)

**Table:**

N1—C1	1.3536 (19)	C7—C12	1.403 (2)
N1—C4	1.3742 (19)	C8—C9	1.390 (2)
N1—H1	0.8600	C8—H8	0.9300
N2—C5	1.288 (2)	C9—C10	1.386 (2)
N2—C7	1.4248 (18)	C9—C13	1.508 (2)
C1—C2	1.346 (2)	C10—C11	1.380 (2)
C1—H1A	0.9300	C10—H10	0.9300
C2—C3	1.407 (2)	C11—C12	1.394 (2)
C2—H2	0.9300	C11—H11	0.9300
C3—C4	1.376 (2)	C12—C14	1.504 (2)
C3—H3	0.9300	C13—H13A	0.9600
C4—C5	1.453 (2)	C13—H13B	0.9600
C5—C6	1.504 (2)	C13—H13C	0.9600
C6—H6A	0.9600	C14—H14A	0.9600
C6—H6B	0.9600	C14—H14B	0.9600
C6—H6C	0.9600	C14—H14C	0.9600
C7—C8	1.388 (2)		
			
C1—N1—C4	109.68 (13)	C7—C8—C9	122.06 (14)
C1—N1—H1	125.2	C7—C8—H8	119.0
C4—N1—H1	125.2	C9—C8—H8	119.0
C5—N2—C7	120.42 (13)	C10—C9—C8	117.65 (15)
C2—C1—N1	108.68 (14)	C10—C9—C13	121.59 (15)
C2—C1—H1A	125.7	C8—C9—C13	120.75 (15)
N1—C1—H1A	125.7	C11—C10—C9	120.68 (15)
C1—C2—C3	107.46 (15)	C11—C10—H10	119.7
C1—C2—H2	126.3	C9—C10—H10	119.7
C3—C2—H2	126.3	C10—C11—C12	122.33 (15)
C4—C3—C2	107.72 (15)	C10—C11—H11	118.8
C4—C3—H3	126.1	C12—C11—H11	118.8
C2—C3—H3	126.1	C11—C12—C7	117.00 (15)
N1—C4—C3	106.46 (13)	C11—C12—C14	122.13 (15)
N1—C4—C5	122.47 (13)	C7—C12—C14	120.86 (14)
C3—C4—C5	131.04 (14)	C9—C13—H13A	109.5
N2—C5—C4	118.40 (13)	C9—C13—H13B	109.5
N2—C5—C6	124.76 (13)	H13A—C13—H13B	109.5
C4—C5—C6	116.83 (13)	C9—C13—H13C	109.5
C5—C6—H6A	109.5	H13A—C13—H13C	109.5
C5—C6—H6B	109.5	H13B—C13—H13C	109.5
H6A—C6—H6B	109.5	C12—C14—H14A	109.5
C5—C6—H6C	109.5	C12—C14—H14B	109.5
H6A—C6—H6C	109.5	H14A—C14—H14B	109.5
H6B—C6—H6C	109.5	C12—C14—H14C	109.5
C8—C7—C12	120.27 (14)	H14A—C14—H14C	109.5
C8—C7—N2	119.97 (13)	H14B—C14—H14C	109.5
C12—C7—N2	119.44 (13)		
			
C4—N1—C1—C2	−0.40 (19)	C5—N2—C7—C12	105.74 (16)
N1—C1—C2—C3	0.5 (2)	C12—C7—C8—C9	−0.7 (2)
C1—C2—C3—C4	−0.4 (2)	N2—C7—C8—C9	−174.23 (13)
C1—N1—C4—C3	0.13 (17)	C7—C8—C9—C10	0.1 (2)
C1—N1—C4—C5	178.29 (13)	C7—C8—C9—C13	−179.93 (15)
C2—C3—C4—N1	0.17 (18)	C8—C9—C10—C11	0.1 (2)
C2—C3—C4—C5	−177.76 (15)	C13—C9—C10—C11	−179.93 (15)
C7—N2—C5—C4	−179.32 (12)	C9—C10—C11—C12	0.5 (3)
C7—N2—C5—C6	0.7 (2)	C10—C11—C12—C7	−1.1 (2)
N1—C4—C5—N2	−3.2 (2)	C10—C11—C12—C14	177.92 (16)
C3—C4—C5—N2	174.48 (16)	C8—C7—C12—C11	1.2 (2)
N1—C4—C5—C6	176.84 (14)	N2—C7—C12—C11	174.73 (13)
C3—C4—C5—C6	−5.5 (2)	C8—C7—C12—C14	−177.81 (15)
C5—N2—C7—C8	−80.71 (18)	N2—C7—C12—C14	−4.3 (2)

### Hydrogen-bond geometry (Å, º)

*Cg1* is the centroid of the C7–C12 ring.

**Table:**

*D*—H···*A*	*D*—H	H···*A*	*D*···*A*	*D*—H···*A*
N1—H1···N2^i^	0.86	2.34	3.1354 (18)	155
C1—H1*A*···*Cg*1^i^	0.93	2.65	3.4298 (16)	142

Symmetry code: (i) −*x*, −*y*+2, −*z*.
